# Parsonage-Turner Syndrome and SARS-CoV-2 Infection: A Literature Review With Case Presentation

**DOI:** 10.7759/cureus.63305

**Published:** 2024-06-27

**Authors:** Androniki Drakou, Pavlos Altsitzioglou, Anastasios G Roustemis, Eleni Vourda, Maria Eleni Papakonstantinou, Spyridon Sioutis, Dimitrios Koulalis

**Affiliations:** 1 Department of Orthopaedics, Laikon Hospital, Athens, GRC; 2 1st Department of Orthopaedic Surgery, National and Kapodistrian University of Athens, Attikon Hospital, Athens, GRC; 3 Department of Allergy and Immunology, National and Kapodistrian University of Athens, Attikon Hospital, Athens, GRC; 4 3rd Department of Paediatrics, National and Kapodistrian University of Athens, Attikon Hospital, Athens, GRC

**Keywords:** parsonage-turner syndrome, immunological mechanisms, covid-19 vaccination, covid-19 infection, neuralgic amyotrophy

## Abstract

Neuralgic amyotrophy, also known as Parsonage-Turner syndrome (PTS), is characterized by severe pain and muscle wasting affecting the anterior body, including the head, shoulder, upper limb, and chest wall. Often triggered by an antecedent event, such as infection, PTS encompasses various conditions historically identified as separate entities. In 1948, Parsonage and Turner unified these conditions under the term neuralgic amyotrophy based on shared features of intense pain and muscular atrophy. Recent studies have highlighted PTS as a spectrum disorder with diverse manifestations, including pure sensory neuropathy, extensive neuropathy, spinal accessory nerve involvement, and diaphragmatic palsy. We reviewed 26 documented cases of PTS following SARS-CoV-2 infection, emphasizing the importance of considering PTS in individuals with a history of COVID-19 due to varied clinical presentations. Standardized diagnostic methods and comprehensive evaluations are crucial for accurate diagnosis and management. Future research should focus on consistent evaluation methods and employing a comprehensive differential diagnosis approach.

## Introduction and background

Neuralgic amyotrophy, also known as Parsonage-Turner syndrome (PTS), is a peripheral nervous system condition characterized by intense pain and significant muscle wasting, primarily affecting the shoulder, upper limb, and chest wall. In most cases, there is an acute identifiable triggering event. Historically, during the mid-1800s, conditions such as serratus magnus paralysis and post-infectious paralysis were recognized. In 1948, Parsonage and Turner identified shared features among these disorders and established neuralgic amyotrophy as a unified entity, marked by intense pain and muscle atrophy. It is now widely acknowledged that these conditions represent different expressions of the same syndrome unified under PTS diagnosis mainly by clinical examination and electromyography (EMG) [1].

## Review

Historically, PTS has been regarded as an uncommon condition, with a yearly occurrence estimated at 1.64 cases per 100,000 individuals [1]. However, the true incidence is significantly greater, as the illness is not adequately acknowledged [2,3]. Research conducted to examine future possibilities reported an incidence rate of 1 per 1,000 individuals [4,5]. There are two main types of PTS, namely, sporadic and inherited. The sporadic form of the condition is more common and usually affects young to middle-aged adults, with an average onset age of 40 years [6]. The genetic variant typically appears around the age of 25 years [4]. PTS can be influenced by genetic and environmental factors. Genetic factors include mutations in the *SEPT9* gene, linked to hereditary neuralgic amyotrophy (HNA), a condition with clinical features similar to PTS. *SEPT9* encodes a protein vital for cytoskeleton organization, essential for nerve integrity and function. While *SEPT9* is the most documented, other genetic loci may also contribute to susceptibility. HNA is inherited in an autosomal dominant pattern, whereas many PTS cases occur sporadically, indicating a combination of genetic predispositions and environmental triggers. Autoimmune mechanisms are implicated in PTS, where the immune system mistakenly attacks the brachial plexus nerves, often following infections, vaccinations, or other immune challenges. Inflammatory cells and cytokines around affected nerves support this hypothesis. Environmental triggers include viral and bacterial infections, such as Epstein-Barr virus or cytomegalovirus, vaccinations, and, occasionally, physical trauma to the shoulder or neck, potentially initiating an inflammatory response​ [6].

Clinical presentation

Van Alfen and colleagues conducted a comprehensive study in the Netherlands on PTS [3]. They described typical clinical features, where PTS was marked by intense neuropathic pain initially, followed by sporadic weakness in the upper limbs, ranging from isolated paralysis of the anterior interosseous nerve to severe bilateral weakness in both arms. Despite variations in presentation, the trio of (i) identifiable triggers, (ii) pain in the upper limb region, and (iii) muscular weakness and wasting in the same anatomical region is unique and aids in diagnosis. Most frequently, PTS is diagnosed based on the two most prominent clinical characteristics, namely, intense pain and muscle weakness or atrophy, which are almost always observed [5]. While localized pain is the primary complaint for most patients, localized sensory loss is infrequent and usually insignificant. Neurological evaluation typically reveals motor system abnormalities [5]. The pain often begins abruptly, waking the patient from sleep or becoming noticeable upon waking up, and progressively intensifies over several hours, prompting swift medical attention. Shoulder or upper extremity movements exacerbate the pain, distinguishing PTS from acute radiculopathies. The pain usually subsides within one to two weeks or transitions to persistent throbbing discomfort, with the rare occurrence of numbness due to cutaneous sensory neuron involvement [6]. Motor impairment and muscular atrophy in the shoulder region emerge after the pain diminishes and the patient starts using the affected limb. Weakness may not be immediately apparent, but muscle atrophy typically develops within a few weeks after onset. In some cases, there may be no weakness or atrophy.

Etiology and pathophysiology

At least half of PTS cases are associated with triggering events, with upper respiratory infections or flu-like illnesses being the most common. Bacterial infections such as *Streptococcus*/*Mycoplasma pneumoniae*, legionnaires’ disease, malaria, typhus, diphtheria, and others, as well as various viruses, including influenza, respiratory syncytial virus, cytomegalovirus, and hepatitis B, have been identified as potential triggers [7]. Several vaccines have been reported as potential triggers for PTS. The most commonly associated vaccine is the influenza vaccine, with cases documented following both seasonal and H1N1 vaccines [7]. The hepatitis B vaccine has also been linked to PTS based on case reports and small case series [8]. Additionally, tetanus toxoid-containing vaccines, such as the DTaP vaccine, have been implicated in a few case reports [9]. Since the rollout of COVID-19 vaccines, there have been reports of PTS following vaccination with various COVID-19 vaccines, including mRNA-based vaccines (Pfizer-BioNTech and Moderna)​ [7]. Additionally, drugs such as nivolumab and botulinum toxin have been reported to induce PTS [8,9].

The hypothesis is that these triggers stimulate the immune system in susceptible individuals, leading to peripheral nerve inflammation characterized by lymphocytic infiltrates and swelling within the nerve [10]. Mechanical strain on the nerves, often due to vigorous upper body activity, may contribute to nerve injury and subsequent inflammation [3]. Repetitive small injuries can lead to increased blood-nerve barrier permeability, allowing immune elements to enter and trigger an autoimmune process [10]. The abrupt onset and single-phase progression of PTS, its association with prior infections, and immunomodulating medications suggest an immune-mediated disease. This is supported by evidence of both humoral and cellular immune responses, including alterations in lymphocyte subsets. PTS patients often show decreased levels of CD3 and increased CD4/CD8 ratios due to decreasing CD8 levels. They also exhibit the presence of antiganglioside and antiperipheral nerve myelin antibodies, as well as terminal complement activation products. Oligoclonal bands are detected in the cerebrospinal fluid (CSF), further indicating immune system involvement [11-14]. Decreased CD3 levels, a marker found on all T lymphocytes, suggest a reduction in the overall T-cell population, reflecting broader immune dysregulation in PTS patients. The CD4/CD8 ratio, an important indicator of immune function, is increased in PTS patients primarily due to a decrease in CD8+ T cells rather than an increase in CD4+ T cells. CD4+ T cells, also known as helper T cells, play a crucial role in coordinating immune responses, while CD8+ T cells, or cytotoxic T cells, are involved in directly killing infected or damaged cells. A reduction in CD8+ T cells can lead to an imbalance in immune regulation, contributing to an autoimmune response against the peripheral nerves. The observed changes in T-cell populations suggest that immune dysregulation is central to the development of PTS, with the decrease in CD8+ T cells impairing the immune system’s ability to regulate inflammatory responses effectively. This dysregulation might result in an inappropriate immune response, targeting components of the brachial plexus and leading to the inflammation and nerve damage characteristic of PTS. The imbalance in T-cell populations might promote an autoimmune environment where the immune system mistakenly attacks nerve tissues, consistent with the hypothesis that PTS is, at least in part, an immune-mediated condition. While changes in T-cell populations are not diagnostic of PTS, they can provide supportive evidence for understanding the underlying mechanisms of the disease [10]. Potential triggers for PTS, such as upper respiratory infections, can also trigger other autoimmune disorders such as acute and chronic inflammatory demyelinating polyradiculoneuropathy [6]. Approximately 10-19% of PTS cases are hereditary, with around 200 documented families worldwide, although race and ethnicity are not mentioned [4,15]. These cases share clinical characteristics with sporadic PTS but may differ in onset age, recurrence rate, and certain physical features [6]. While both hereditary and sporadic forms usually occur in individuals in their 30s and 40s, the hereditary type may present more frequently in children [6]. Hereditary PTS is inherited in an autosomal dominant manner, with mutations in the *SEPT9* gene on chromosome 17q accounting for about 55% of cases in North American families [10,16,17]. However, the genetic anomaly in the remaining 45% of families remains unidentified, indicating heterogeneity in hereditary PTS.

Electrodiagnostic studies show that both forms of PTS primarily involve axonal breakdown and subsequent Wallerian degeneration, leading to conduction failure and muscle atrophy. Nerve conduction studies (NCS) assess the function of peripheral nerves by measuring the speed of nerve impulses. In PTS, NCS may show abnormalities such as decreased compound muscle action potentials (CMAPs) or conduction blocks in affected nerves, indicating nerve damage or dysfunction. Needle EMG, which involves inserting a fine needle electrode into muscles to evaluate their electrical activity at rest and during voluntary contraction, can reveal specific abnormalities in PTS. These include denervation, characterized by spontaneous fibrillation potentials and positive sharp waves in affected muscles; reinnervation, indicated by polyphasic motor unit potentials (MUPs) as a sign of ongoing recovery in muscles; asymmetry, highlighted by comparing the affected and unaffected sides to identify differences in muscle involvement; and the pattern of involvement, which can vary and aids in the localization and diagnosis of PTS [11]. Localized demyelination is rare, occurring in fewer than 1% of cases [6].

Diagnostic workup

Laboratory investigations have limited diagnostic utility in PTS, primarily aiding in detecting specific illnesses associated with its development and assisting in certain differential diagnoses [10]. Electrodiagnostic studies, including needle EMG and NCS, are commonly used for diagnosis [18]. EMG is valuable for identifying muscle denervation, although complete denervation may take up to four weeks to manifest, limiting its early diagnostic usefulness [18]. NCS provides information on nerve damage location, but during subacute stages with some reinnervation, nerve parameters may appear normal, reducing effectiveness [10]. Identifying nerve conduction slowing or blockage in certain nerves can be challenging due to their anatomical location, and 80% of clinically damaged nerves may not show sensory anomalies on NCS [19]. NCS may reveal conduction abnormalities, such as conduction block or slowing, in nerves innervating affected muscles. These findings suggest focal demyelination or axonal injury within the brachial plexus, supporting the diagnosis of neuralgic amyotrophy. Decreased CMAP amplitudes, typically greater than 5 mV in healthy individuals, may be observed in muscles supplied by affected nerves, indicating axonal loss or dysfunction. This finding is consistent with the motor deficits seen in PTS and helps localize the site of nerve involvement. EMG typically shows spontaneous activity at rest, such as fibrillation potentials (small, polyphasic potentials of low amplitude (<100 µV) and short duration (<10 ms)) and positive sharp waves (triphasic, high-frequency potentials), indicating ongoing denervation. These findings are indicative of axonal injury or loss within the affected nerves and support the diagnosis of PTS. During the recovery phase, EMG may reveal polyphasic MUPs, which are complex waveforms with increased duration and amplitude, reflecting ongoing reinnervation attempts. This pattern suggests regenerative changes and is characteristic of the recovery process in PTS. Additionally, EMG studies may demonstrate muscle atrophy and reduced recruitment of motor units in affected muscles, correlating with the clinical presentation of weakness and functional impairment. While these abnormalities are suggestive of PTS, it is important to consider other conditions that can cause similar EMG and NCS findings, such as compressive neuropathies, radiculopathies, or motor neuron diseases. Clinical correlation with the presentation, imaging studies, and exclusion of other potential causes are essential for an accurate diagnosis. In PTS, conduction velocity may be normal or mildly slowed in affected nerves, reflecting focal demyelination or axonal injury within the brachial plexus, with normal conduction velocity typically being greater than 50 m/s in upper extremity nerves. Abnormal findings alone are not diagnostic of PTS and may overlap with other conditions, underscoring the importance of comprehensive evaluation [12]. Therefore, a normal NCS does not rule out PTS [6]. When both the long thoracic nerve and left suprascapular nerve are paralyzed simultaneously, alternative diagnoses such as cervical root or shoulder joint problems are less likely [9]. Mononeuropathies may be due to entrapment neuropathy, synovial cysts, or lipomas. Other possible diagnoses include meningoradiculitis, neoplastic plexopathy, or vasculitis based on clinical and further studies. If pain is absent, potential diagnoses include chronic idiopathic demyelinating polyneuropathy, multifocal motor neuropathy, Lewis-Sumner syndrome, hereditary neuropathy with vulnerability to pressure palsy, and facio-scapulo-humeral myopathy [7]. Additionally, PTS has been observed as a consequence of SARS-CoV-2 infection [13].

Literature review

We studied 21 articles that provided information on 26 cases of patients. The cases are from various countries, and the patients’ ages ranged from 17 to 76 years, with the majority being males (21 out of 26, or 80.8%). The publication dates spanned from 2020 to 2022.

Patients’ Characteristics

Regarding patients’ immunological status, one report mentioned a patient receiving a vaccination a few months before PTS [20], without specifying the vaccine or the date of vaccination. Another patient received the primary vaccination with the Moderna mRNA 1273 vaccine four months before PTS [21], while two patients had not been recently vaccinated [22,23]. These patients were previously healthy. One patient underwent kidney transplantation for polycystic kidney disease and was on prednisolone, tacrolimus, and mycophenolate mofetil [24]. In another study, both siblings had a history of steroid allergy, as stated by the reviewer [25]. Two patients were examined for autoimmune disorders, but vaccination status was not provided [26]. Vaccination status or immunosuppression details were not provided for the remaining 17 out of 26 (65.4%) PTS cases. Nine (34.6%) patients had notable comorbidities, including clavicle fracture [27], familial history of PTS [25], work-related shoulder pain history [28], shoulder arthroplasty [28], psoriatic arthropathy in remission [29], anterior shoulder dislocation episode [20], or prior rotator cuff repair performed 2.5 years ago [30]. All cases (26/26, 100%) experienced a sudden onset of PTS. The onset timing of PTS was uncertain in two out of 26 (7.7%) patients [14,15]. Neurological symptoms were observed in 20.8% of patients (5/24) at the same time as their COVID-19 diagnosis [16-19]. In 12.5% of cases (3/24), these symptoms appeared within one week of the infection [20-22]. Nine (37.5%) patients experienced PTS after one month of infection, specifically after more than seven days [18,23-29]. Among the patients, PTS symptoms manifested after a duration exceeding one month following their COVID-19 infection in 29.2% of cases [5,18,30-33]. The onset of PTS was not clarified in two patients.

The sensation of pain was reported in the majority of instances (25/26, 96.2%). Of the total patients (1/26, 3.8%) admitted to the intensive care unit (ICU) with acute respiratory distress syndrome (ARDS), only one had neuralgic amyotrophy that affected the C5-C6 nerve roots, as well as the lateral pectoral and phrenic nerves. This patient experienced reduced sensitivity to touch, muscle weakness, muscle loss, and paralysis of the diaphragm [29]. Of the 26 patients, 21 (80.8%) exhibited a motor deficiency [5,14-16,18,20,24-34]. Of the 26 patients, muscle strength was normal (5/5) in three (11.5%) patients. For assessing muscle strength, the Oxford muscle strength scale was used [35]. Two patients had a pure sensory PTS condition, while one case was not clear. The investigators first reported normal motor function in this case, but eight weeks later, the patient developed scapula winging. Furthermore, the authors failed to describe any motor impairment in two familial cases (2/26). However, the patients did exhibit muscle atrophy in the deltoid, supraspinatus, and scapular muscles [17].

Overall, 14 out of 26 patients (53.8%) experienced muscle atrophy [5,14-18,18,21,22,24,32]. Nevertheless, nine out of 26 patients (34.6%) reported experiencing a loss of sensory function [14,19,22,23,25,26,28-30]. Of the 26 patients, NCS were conducted in 11 (42.3%) cases [5,19,22-24,26-28,32], while EMG was performed in 18 (69.2%) cases [5,16,18,19,21-30]. The results of the NCS differed based on the specific nerve fibers that were impacted and the date of the test. The authors documented the presence of acute motor axon loss indicators [14], normal latencies with a significant decrease in CMAP amplitude [5], evidence of subacute plexopathy [24], and normal results three months after the onset of PTS [26]. Furthermore, a small number of patients exhibited a lack of sensory responses [14] or reduced amplitude of sensory nerve activation potential [23]. Several authors conducted electrophysiological tests on a total of four patients [14,15,17]. Similarly, the EMG findings varied: indications of nerve damage [5,15,18,21,27], sporadic nerve network dysfunction [31], presence of sharp waves and fibrillation [24], long-term nerve network dysfunction with nerve regrowth [32], and normal results five weeks after the onset of PTS symptoms [23]. A neuromuscular ultrasonography investigation was used in 19.2% (5/26) cases [15,21,23,28,29]. The results revealed that the damaged nerves showed an increase in size [15,21,28], several areas of nerve injury [15], and muscle wasting [29]. A case of post-COVID-19 pure sensory neuralgic amyotrophy involved severe pain and sensory deficits in the upper limb. Despite normal muscle strength and reflexes, electrodiagnostic testing revealed nerve impairment. This highlights the potential of COVID-19 to trigger neuralgic amyotrophy and the importance of timely diagnosis [23].

A severe COVID-19 case led to persistent dyspnea, left shoulder motor deficit, and respiratory failure, diagnosed as neuralgic amyotrophy affecting diaphragmatic nerves. Persistent respiratory symptoms post-COVID-19 should prompt evaluation for diaphragmatic causes, emphasizing the need for comprehensive respiratory assessments in such patients [29]. MRI was conducted in 17 out of 26 cases, accounting for 65.4% of the total. The predominant observation was the presence of muscle edema in 10 out of 17 cases, accounting for 58.8% of the cases [14-16,22,28-30,32,33]. This was followed by hyperintensity of the afflicted fascicles, which was observed in eight out of 17 cases, accounting for 47.1% of the cases [15,19,20,26,27,29,31]. Muscle atrophy was observed in five out of 17 patients, accounting for 29.4% of the cases [5,16,29,30]. One patient [28] exhibited an increase in the visibility of the damaged nerve due to contrast enhancement. Furthermore, the MRI diffusion neurography of the brachial plexus revealed that the anisotropic fractions and apparent diffusion coefficient (ADC) exhibited a tendency toward isotropy when compared to the unaffected side. This indicates a structural disorder of the neural elements, which is consistent with PTS. No areas of increased density were seen when contrast media was used. According to the investigators, the signal asymmetry was likely caused by inflammation [24]. Other authors observed a hyperintense signal on the affected nerve bundles using a technique called short inversion time inversion recovery. This signal does not indicate any abnormality in the size or presence of a lesion, as detected by MRI. Similarly, segmental diffusion-weighted imaging did not show any restriction in the diffusion of the nerve trunk, and the corresponding ADC also showed a low signal. Furthermore, a single instance of hourglass constrictions was identified [15]. Out of these two patients in this study, only one underwent a nerve biopsy and why the other did not was not stated by the authors. The biopsy was conducted on the brachial cutaneous nerve, revealing significant axonal damage [31].

Treatment

Initially, non-steroidal anti-inflammatory drugs (NSAIDs) were prescribed for 30.8% (8/26) of cases, although they had low or no impact [14,17,21,26,30,32]. Additionally, muscle relaxers were administered to two individuals, accounting for 7.7% (2/26) of the total. According to other investigators, a small number of patients (2/26, 7%) were given acetaminophen initially, although it provided only limited alleviation [22,23]. Of the patients who had significant pain, 19.2% (5/26) were given gabapentin or similar drugs that modulate the nervous system, such as gabapentin-type neuromodulators [17,19,21,26]. Pregabalin was administered to three patients (11.5%) [22,24,26], while duloxetine was prescribed to a single patient [19]. Of the total patients, five individuals (representing 19.2% of the sample) received treatment with opioids [14,17,22,30].

Furthermore, a single patient had local administration of steroids and lidocaine via injections, resulting in a minor amelioration [22]. Two patients were administered analgesic medicine; however, the authors did not explicitly state the exact classification of the drugs (2/26, 7.7%) [5]. Corticosteroids were given to 46.2% of the PTS cases (12/26 instances) [5,14-16,18,19,22,25,27,28,33]. A single patient was administered intravenous methylprednisolone at a daily dosage of 1,000 mg for five days with no information on the hypothalamic-hypophyseal-adrenal axis. However, the treatment was discontinued due to dermatological adverse effects. The treatment included a regimen of intravenous immunoglobulins (IVIG) at a dosage of 25 g per day for five days. This treatment provided some relief from pain but did not result in any increase in muscle strength [22]. The remaining patients were administered oral steroids. The authors reported the outcome in eight cases among patients who underwent steroid therapy. A single patient, who experienced only musculocutaneous involvement, achieved complete recovery within one month [15]. Another patient with PTS underwent a three-week course of oral prednisolone (25 mg), followed by a gradual reduction in dosage. After 21 days of starting the treatment, the patient showed some improvement in muscle strength, but not complete recovery. During the two-month follow-up, the patient’s shoulder examination yielded normal results, indicating the absence of pain or functional restrictions [25]. Four patients exhibited partial amelioration at subsequent appointments at varying time intervals (ranging from two months to six months) [16,18,19,27]. Neurological examination findings in a single person remained unchanged after eight weeks [22]. Another instance documented alleviation of pain at seven- and 14-day intervals, although the evaluations of motor and sensory functions were the same [28]. The authors administered extended-release pirfenidone to two patients, a brother and sister, who had a history of steroid allergy. The treatment began on day 22, taking into account the potential anti-inflammatory effects of the medication [17]. By the 26th day of infection, the symptoms of neuralgia had diminished [17]. Rehabilitation was advised for 42.3% of PTS patients (11/26 patients) [5,16,18,24,26,29,30]. Of the 26 patients with pure motor PTS, one patient (3.8%) did not receive any treatment. However, the evolution was positive, as all symptoms completely disappeared after three months [27]. No information regarding the treatment was provided by some studies [20,31].

Follow-Up

No information on the progression of PTS was available for seven out of 26 patients (26.9%) [5,14,18,29,31,33]. Of the 19 patients whose outcomes were recorded, 5/19 (26.3%) experienced a full disappearance of symptoms at different follow-up visits: one month [15], two months [25], three months [14,27], and six months [24]. Of 19 patients, 11 (57.9%) showed improvement in clinical evaluation. Follow-up visits occurred at various times: 13 days [21], approximately three weeks [25], six weeks [22], two months [27,33], three months [29], four months [28,35], six months [20], eight months [30], and nine months [28].

Parsonage-Turner Syndrome in Children

PTS is an extremely rare entity in the pediatric population. The precise etiology is uncertain and it may manifest as early as the first week of life. A triggering event usually precedes disease manifestation, most commonly an infection, recent vaccination, or trauma. It is hypothesized that the triggering event itself injures the brachial plexus or the injury is due to an abnormal response of the immune system [36]. In children, PTS may present with a broad range of symptoms. Classically, it initiates with acute neurologic pain, described as sharp, throbbing, in the shoulder area not responding to usual analgesic treatment. As the pain wanes, paresis ensues followed by muscular atrophy. Involvement of the contralateral side is infrequent but may occur. Uncommon presentations include peripheral nerve involvement and absence of pain. Contrary to adults where the pain is almost invariably present, strikingly, one-third of children may present without pain, complicating the diagnosis [37]. Regarding treatment, a consensus has not yet been reached. Scarce available data argue over early corticosteroid use. Even more limited evidence shows that the use of IVIG improves outcomes. Analgesics may be used to relieve pain. Despite that, PTS in children has a better prognosis than in adults. The majority of patients recover, albeit slowly sometimes [38].

SARS-CoV-2 Infection

COVID-19 diagnosis was confirmed through positive real-time reverse-transcription polymerase chain reaction (RT-PCR) tests in 15 out of 26 patients, representing 73.1% of total cases [20,22,24-27,33-37,39]. However, the cycle threshold (Ct) value of the positive RT-PCR test was not reported in any of the cases. For nine out of 26 patients (34.6%), information on how the COVID-19 diagnosis was established was not provided [21,28-32,38]. Two (7.7%) patients did not undergo RT-PCR testing, and the diagnosis of SARS-CoV-2 infection was determined retrospectively. One patient showed anti-SARS-CoV-2 antibodies, indicating a previous infection or exposure, with high levels of IgG and normal levels of IgM [23]. In another case, a patient initially tested negative for the RT-PCR test but tested positive for IgG antibodies a few weeks after experiencing a respiratory infection [40]. Regarding the severity of COVID-19, 15.4% of the patients (4/26) had mild disease, 3.8% (1/26) had moderate-to-severe infection, and 34.6% (9/26) had severe illness. The severity of the SARS-CoV-2 infection was not documented in 46.2% of cases on [17,18,20,22-24,27,33]. Of the patients diagnosed with severe COVID-19, 30.8% (8/26) needed ICU admission [5,14,15,18,29,31,32]. Of these eight patients, four (50%) experienced PTS symptoms either a few days or weeks after being discharged from the ICU or after being removed from a ventilator [5,14,18,32].

Case description

**Figure 1:**
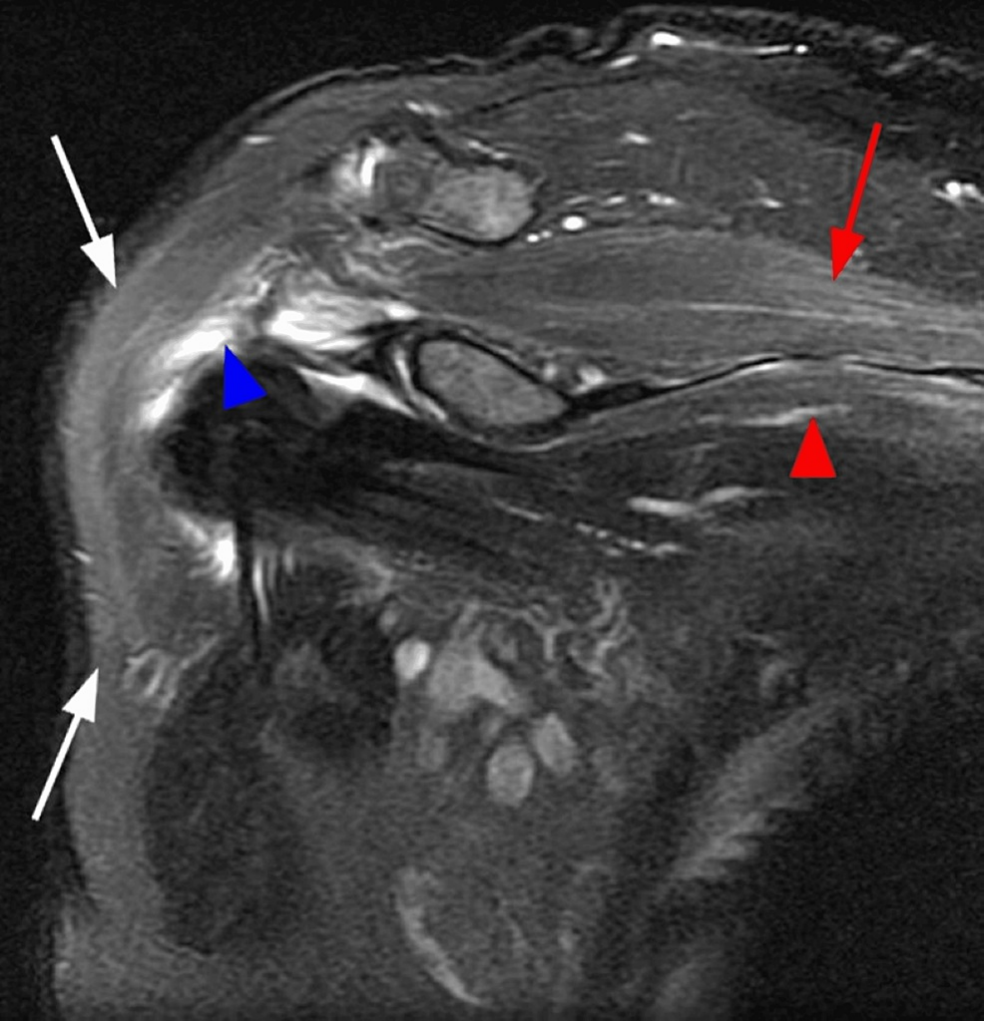
In the T2 fat suppression sequence at the sternal level, mild heterogeneous edema is evident in the supraspinatus muscle (red arrow) and the upper part of the subscapularis (red triangle). The edema in the subscapularis is presumed to involve the participation of the upper subscapular nerve originating from C5 and C6 or from peripheral collateral nerves. The non-homogeneous distribution of fat (white arrows) is observed, which blends with the signal of the deltoid. The blue arrows indicate subacromial-subdeltoid bursa fluid. Limited passive movement is not characteristic of Parsonage-Turner syndrome and other diagnoses should be considered (e.g., adhesive capsulitis).

**Figure 2:**
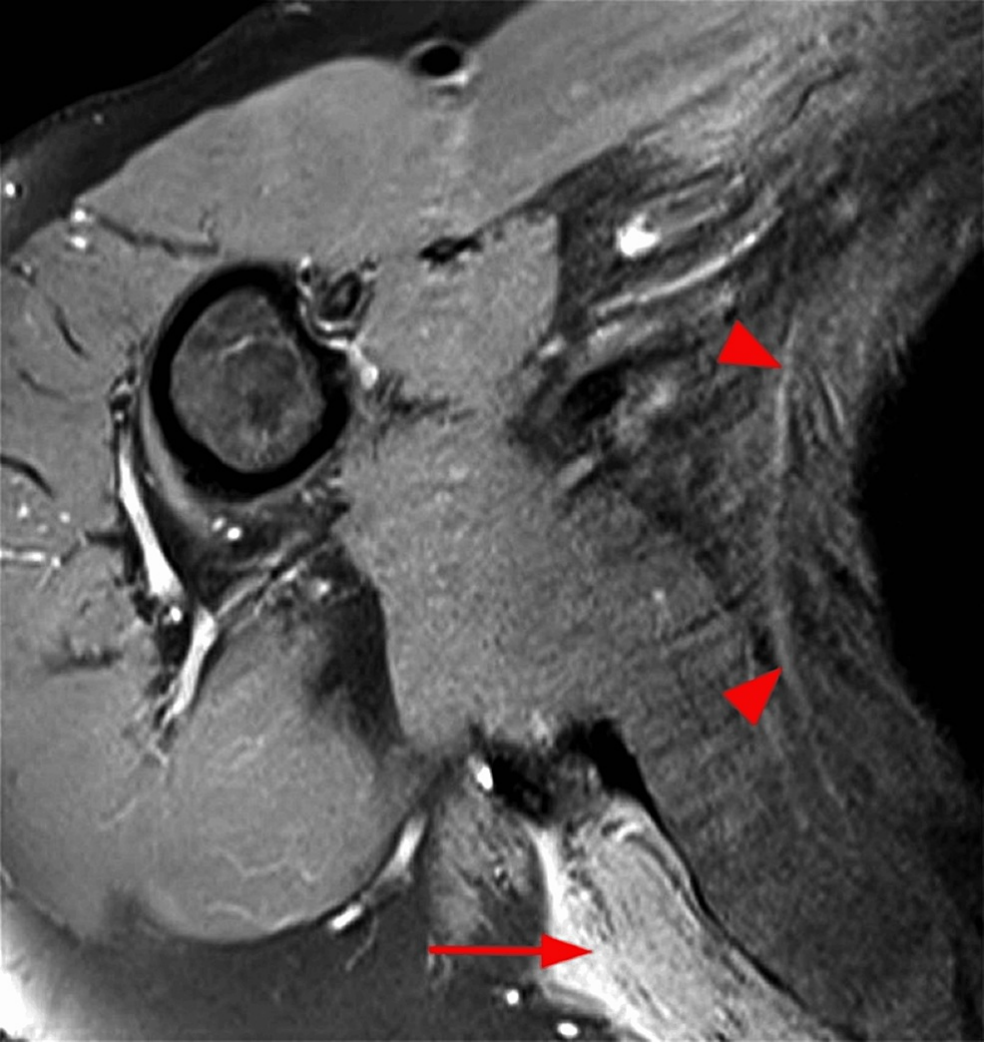
At the transverse level, depicted with fat suppression from the same patient, there appears a linear and high-frequency signal in a relatively thin portion of the anterior serratus muscle (triangles), which is not ideally visualized due to the presence of various artifacts. Swelling of the subacromial area is again observed (arrow).

**Figure 3:**
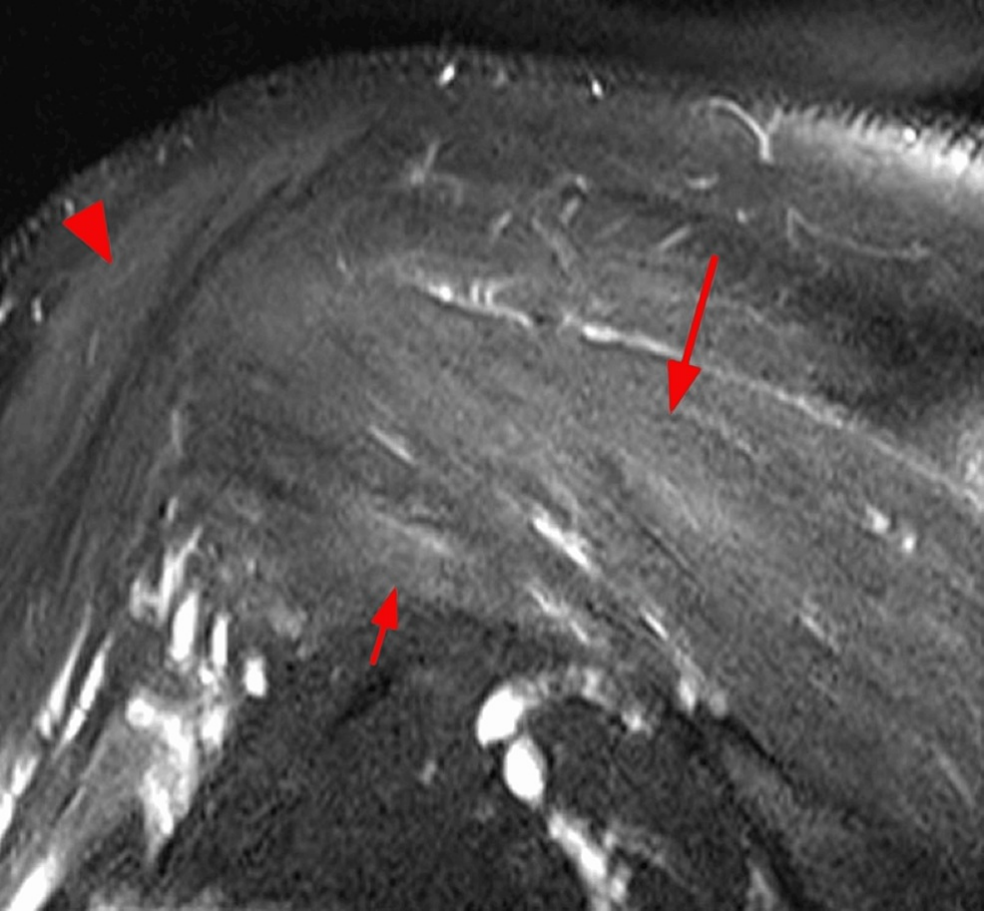
Heterogeneous edema is evident in the infraspinatus (red arrow), the deltoid muscle (red triangle), and the teres minor.

We present a case of PTS following COVID-19 vaccination in a right-handed 65-year-old male without any other pathology. The patient received the first dose of the Pfizer/Biotech mRNA vaccine for COVID-19, without presenting clinically significant side effects, except for transient malaise and fatigue. Six weeks after vaccination, he developed intense and continuous pain in the shoulder girdle accompanied by muscle weakness, which did not improve with conservative measures such as rest, warmth, or anti-inflammatory treatment. Later, the patient noticed reduced strength and dexterity in his right hand. EMG was performed, along with a study of electrical conductivity, as well as an MRI of the brachial plexus. The EMG revealed signs of denervation in the right upper limb, supported by MRI findings, which showed mild swelling and muscle inflammation (Figures 1-3). The patient improved after receiving treatment with corticosteroids and intensive physiotherapy.

Discussion

This study detected a total of 26 cases of PTS occurring after infection with the SARS-CoV-2 virus. The cohort examined by van Alfen et al. comprised 67.5% of males [4]. Nevertheless, as PTS is believed to have an autoimmune etiology, it is anticipated to exhibit a higher prevalence in females. Hence, a yet unidentified component specific to sex in this condition may render male patients more susceptible to episodes [4]. Furthermore, two cases had a family history, as reported in reference [17], but genetic testing was not conducted. The incidence of this fraction (7.7%) is lower than that reported in the general population (19%). However, it is worth noting that numerous studies did not provide information on the family history of their patients. Among the patients in our study, 15 out of 26 (57.7%) reported experiencing severe discomfort. Nevertheless, the degree of pain severity was not explicitly indicated in 10 out of 26 instances, accounting for 38.5% of the total. One patient exhibited an absence of pain [29]. Of the 16 individuals for whom pain intensity data was available, 93.8% experienced intense pain. This data has similarities to earlier reports documented in the literature [11,39]. Typically, the pain in PTS arises within a few hours, and in the majority of cases, the episodes commence throughout the night [11,40]. Nevertheless, the majority of studies in our review lacked comprehensive patient pain data. Of the 25 patients who experienced pain, 22 (88%) had symptoms on one side only, whereas three (12%) had symptoms on both sides in an uneven pattern [14,22,33]. The prevalence of unilateral pain in our patient population exceeds that reported by other studies on individuals with PTS. For instance, van Alfen et al. found that 71.5% of patients experienced discomfort on one side, whereas 28.5% had pain on both sides [11]. Among five out of 25 patients (20%), the presence of pain resulted in sleep disruptions [14,18,26,30]. Our evaluation found a significantly lower percentage (93.5%) compared to what has been reported in the literature [11]. This discrepancy may be attributed to the fact that the authors did not include any information regarding sleep quality in 20 out of 25 cases (80%). Out of the 25 patients (four males and one female), 20% (5/25) reported a heightened mechanical sensitivity, which refers to experiencing pain when the affected limb is moved, pressed, or touched [18,22,26,28,33]. In a single instance (1/25, 4%), the discomfort was neither relieved nor intensified by shoulder mobility [30]. However, these features were absent in the majority of cases (19/25, 76%). During the incident, numerous people with PTS experienced three distinct pain phases. The persistent first pain may be succeeded by severe neuropathic stabbing or shooting pains, frequently triggered by movement, lying, or prolonged limb positioning. Approximately 66% of individuals with PTS have ongoing musculoskeletal pain following their first diagnosis. This subsequent form of pain typically occurs in the specific location where the paretic or compensating muscles originate or attach, mainly in the periscapular, cervical, and occipital regions [11]. Unfortunately, the studies included in this analysis did not provide a comprehensive description of the patient’s symptoms. The temporal sequence of other symptoms and indications that occur after the pain was documented in eight out of 25 patients (32%) [18,21,22,24,27,28,30,33]. Out of the total, 75% (6/8) experienced further symptoms after two weeks. In 25% (2/8) of patients, the motor deficiency was observed after a longer period, either seven weeks [24] or a few weeks [18]. Our findings align with previous studies, in which researchers discovered that 27.2% of all instances of paresis did not become apparent until more than two weeks following the initial beginning of discomfort [11]. An extensive examination of individuals with PTS revealed that approximately one-third of these patients have a gradual worsening of their motor impairment over varying periods: 8.6% over days, 16% over weeks, and 5.6% over months [11]. Nevertheless, we did not discover any documented instances of the exacerbation of the motor impairment. Of the 24 patients who had a motor deficit or muscle wasting, the motor deficit was evaluated using the Medical Research Council (MRC) grading method in 10 patients. The intensity ranged from 1/5 to 4/5, with 70% of people showing a maximal deficit of 3/5 or 4/5 [15,16,18,20,27,32], while 30% of cases exhibited severe paralysis with an MRC of 1/5 or 2/5 [18,22,24]. In contrast, the research indicates that approximately two-thirds of patients exhibited significant motor impairments [11]. During our evaluation, 26.3% of patients who provided information about their results experienced a full recovery. Furthermore, the clinical evaluation demonstrated that 53.8% of patients exhibited muscular atrophy, whereas existing literature suggests higher proportions ranging from 75.4% to 88.5% [11]. This discrepancy may be attributed to the timing of the examination, as previous research has indicated that atrophy typically becomes noticeable after approximately five weeks. Alternatively, it could be attributable to the fact that the majority of individuals in our study did not exhibit a severe motor deficiency. In addition, the presence of muscle weakness and atrophy may not be easily detected. Initially, weakness may only be noticeable in the early stages of PTS if the patient’s movements are restricted due to discomfort. In addition, synergistic muscles can conceal the motor impairment, or a muscle on top may conceal the muscle degeneration (e.g., the trapezius muscle conceals the atrophy of the supraspinatus muscle, and the biceps muscle may conceal the atrophy of the brachialis muscle) [6]. The sensory symptoms observed in our series deviate from the findings of other studies that have examined PTS. Paresthesia was observed in 46.2% of the PTS people (12/26) [14,17,18,20-22,26,28,31,32]. Nevertheless, 34.6% of the patients (9/26) experienced a sensory loss [14,19,22,23,25,26,28-30]. The sensory symptoms and indicators were observed either independently or in conjunction with motor findings, exhibiting various patterns. While prior studies have shown that over 50% of patients with PTS do not experience any improvement during follow-up, there is a lack of detailed information regarding the progression of sensory symptoms in PTS patients after being infected with SARS-CoV-2. The authors of these studies primarily focused on the recovery of motor function. Furthermore, our analysis did not find any instances of autonomic nervous system involvement among the patients, such as vegetative and trophic skin changes, edema, or temperature dysregulation. However, it is worth noting that these symptoms have been described in 15.4% of patients with PTS [11]. Several individuals had involvement of nerves beyond the brachial plexus, such as the lumbosacral plexus [32], phrenic nerve [29], and spinal accessory nerve [5]. Hereditary types of PTS may occasionally impact the lumbosacral nerves, as indicated by previous studies [39]. However, investigations on a significant number of sporadic cases of PTS did not observe any instances of muscle involvement in the lower extremities [41]. Moreover, if the weakness in the forequarter region does not occur at the same time as the involvement of the lower extremity muscles, it is unclear whether they are related to the same illness [39]. Both lumbosacral radiculoplexus neuropathy and PTS exhibit identical clinical characteristics, including intense pain, muscular weakness, and atrophy. In the present review, the patient with extended PTS did not undergo genetic testing. However, the authors observed that he had no family history of neurological illnesses [32]. Diagnosing phrenic nerve involvement is a challenge. In a study examining phrenic neuropathies caused by PTS, 10 out of the 17 cases were found to be isolated, indicating no involvement of other concurrent nerves [42]. The patients may exhibit either unilateral or bilateral diaphragmatic paralysis. If it is unilateral, it may go undiagnosed. Moreover, when phrenic neuropathies occur in isolation, they are prone to being undetected if the individual does not experience any symptoms or if they just experience slight and temporary difficulty in breathing. Diagnosis is more probable when there is a preceding incident or intense shoulder pain [39]. In the current analysis, a single patient exhibited phrenic nerve involvement [29]; however, the potential occurrence of diaphragmatic palsy may have been disregarded. The occurrence of cranial nerve involvement ranges from 0% [43] to 10% [44], with a higher prevalence in individuals with genetic types of PTS [11]. According to research on sporadic PTS, the cranial nerve most frequently affected was the spinal accessory nerve, which accounted for around 2% of all lesions [41]. Two individuals in our analysis exhibited spinal accessory nerve damage [5]; however, genetic testing was not conducted and there was no mention of their family history. Pulmonary imaging, such as chest X-ray or CT, revealed abnormalities in nine out of 26 patients, accounting for 34.6% of the total. These imaging techniques aid in examining the differential diagnosis for PTS (such as Pancoast tumor) and the potential presence of diaphragmatic paralysis. Nevertheless, in our specific instances, the procedure was mainly conducted for COVID-19, with just a few exceptional occurrences [5,29]. In addition, 30.8% of the patients (8/26) underwent an MRI of the cervical spine to rule out any spinal abnormalities [20,21,25-28,30,32,45-51]. Shoulder MRIs were conducted in seven out of 26 patients, accounting for 26.9% of the total cases [5,18,24,33]. Although several patients exhibited alterations on the cervical spine MRI, the findings did not elucidate the patient’s clinical presentation and progression. The predominant diagnostic test utilized was EMG, accounting for 69.2% of instances. This was followed by an MRI of the upper limb and brachial plexus, which was employed in 65.4% of individuals. NCS were conducted in 42.3% of patients, while neuromuscular ultrasonography was employed in 19.2% of cases. The authors conducted a nerve biopsy in only one instance, which accounted for 3.8% of all cases. Remarkably, the MRI scan yielded abnormal results in 16 out of 17 patients, which accounted for 94.1% of the total. Only one instance of MRI without gadolinium exhibited normal results [25]. The diagnosis of PTS typically relies on clinical history and neurological examinations, although electrodiagnostic studies are also beneficial. These studies can identify specific lesions in the peripheral nervous system and recognize common patterns such as mononeuropathies and multiple mononeuropathies, which primarily affect motor nerves, causing significant damage to one muscle while sparing others. However, NCS does not definitively rule out PTS. MRI and ultrasound examinations provide further insights into specific abnormalities [48-51]. While MRI is more useful for imaging the brachial plexus, ultrasound is valuable for extraplexal imaging due to its ability to accurately track nerve paths and fascicles. As most PTS lesions are outside the nerve plexus, ultrasound has advantages over MRI, including enhanced spatial resolution, lower costs, simplicity in making side-to-side comparisons, and real-time examination capabilities [52]. While specific blood, urine, or CSF tests for diagnosing PTS are unavailable, routine blood work is necessary to rule out urgent and treatable diseases. Metabolic investigations may indicate elevated liver enzymes, warranting a comprehensive hepatitis profile. Serological analysis for common infections and laboratory examination for vasculitis may also be required. Ancillary studies relating to certain disorders, such as human immunodeficiency virus infection, are beneficial when patients exhibit risk factors for these conditions. According to recent data, 26% of the individuals evaluated for PTS have been shown to have antiganglioside antibodies [11]. However, in this review, the authors examined the antiganglioside antibodies in just one patient [27]. Managing pain is crucial during the acute period of PTS. Typically, NSAIDs and acetaminophen are not effective in relieving pain [39]. Instead, it is recommended to use medication specifically designed for neuropathic pain, such as antiepileptic drugs such as gabapentin or pregabalin, or tricyclic agents such as amitriptyline or nortriptyline [39]. Patients experiencing PTS after being infected with SARS-CoV-2 were administered different types of analgesic drugs to alleviate discomfort. Overall, 46.2% of PTS cases received corticosteroids in various doses and schedules, yielding varied outcomes ranging from complete recovery within one month to partial symptom improvement or no improvement. However, it is necessary to conduct randomized placebo-controlled trials to assess the impact of corticosteroids and other drugs on individuals with post-traumatic stress. A single patient suffering from PTS after being infected with SARS-CoV-2 underwent a treatment of IVIG. The patient experienced some reduction in discomfort but did not observe any improvement in muscle strength [22]. A limited number of studies on IVIG treatment indicate that initiating treatment early may result in a shorter duration of the disease, proving to be more effective compared to delayed treatment [45]. However, additional investigation is necessary in this area. Physical therapy was advised in 42.3% of the patients examined in the current analysis. During the acute phase, the pain is intense and might be worsened by movement of the leg. Consequently, it is advisable to initiate physical treatment as soon as the discomfort allows for mobility. This should include exercises that improve the range of motion, stretching exercises, strengthening of the agonist muscles, and the use of orthotic devices [39]. Surgical surgery is only considered for patients with persistent or severe illness who have not responded to non-invasive treatment. Nevertheless, it is recommended to wait for a minimum of three months to allow for any natural healing process [46-48]. However, if no obstructions were seen during additional examinations, it is advisable to continue with conservative treatment [49]. The facts about the prognosis of PTS exhibit variability. For instance, several studies discovered that 36% of patients experience recovery after one year, 75% within two years, and 89% within three years [43]. According to other research, only 11 out of 83 patients with PTS experienced full recovery throughout a 17-year period of observation [50]. However, it is important to assess the probability of healing for each distinct injury by applying the fundamental principles of reinnervation [39]. Among those who had previously contracted SARS-CoV-2, 26.3% experienced a full recovery from PTS symptoms within six months.

Limitations

The limitations of this evaluation mostly pertain to the quality of the studies that were included. The process of extracting data was difficult because of the absence, incompleteness, or ambiguity of the descriptions. This may be attributed to the absence of a consistent approach and explicit reporting criteria, which leads to significant methodological variance in studies on SARS-CoV-2. In addition, certain studies included in the research sparked intriguing scientific discussions [51-54]. Furthermore, there is a heightened likelihood of bias linked to case reports, and the restricted inferences they offer may give rise to issues. The limitations of our findings are determined by the quality and scope of information provided in the included reports, which varied among the 26 patients included in the study. This pertains to both the PTS and the severe ARDS of SARS-CoV-2 infection. Information regarding past COVID-19 is limited in the majority of individuals. Furthermore, the assessment of other potential causes for PTS, such as coinfections, intravenous procedures (such as intravenous therapy, contrast administration, or blood withdrawal), and specific drugs, is incomplete. Nevertheless, case reports serve as a suitable study design and play a crucial role in furthering research, especially for uncommon medical problems. Although there are limitations in the methodology, studying individual patients offers valuable insights into the causes, development, progression, and treatments of a disease [55]. They have a crucial function in illuminating novel occurrences and offer initial evidence to further examine theories using statistical methods. It is important to consistently evaluate factors such as the initial features of the patients, progression of the disease, and treatments in all investigations. Furthermore, it is crucial to utilize a comprehensive differential diagnosis.

## Conclusions

This is the initial comprehensive analysis of PTS after SARS-CoV-2 infection based on the most up-to-date information available. So far, we have identified 26 documented cases, each with different clinical and paraclinical observations. The clinical and paraclinical presentations varied, encompassing classical PTS, pure sensory neuropathy, extensive neuropathy, involvement of the spinal auxiliary nerve, and diaphragmatic palsy. In addition, two cases involving family members were reported. This review emphasizes the importance of maintaining a high level of suspicion for PTS in patients who have previously been infected with SARS-CoV-2. This is because the clinical symptoms of PTS may vary.

However, it is necessary to have a defined methodology to examine and document PTS. Subsequent investigations should strive to conduct a thorough evaluation of individuals. It is important to consistently evaluate factors such as patient characteristics, disease progression, and therapy in all investigations. Furthermore, it is crucial to utilize a comprehensive differential diagnosis.
